# Disseminated tuberculosis with rare coccygeal involvement: a case report

**DOI:** 10.1099/acmi.0.000924.v3

**Published:** 2025-06-30

**Authors:** Sarra Baziaa, Adil Zegmout, Mohamed Beaouiss, Soufiane El Fathi, Aniss Rafik, Hicham Souhi, Ismail Rhorfi, Hanane El Ouazzani

**Affiliations:** 1 Pulmonology Department, Mohamed V Military Hospital, Rabat, Morocco

**Keywords:** disseminated tuberculosis, elbow joint tuberculosis, immunocompetent, polymerase chain reaction (PCR), sacrococcygeal tuberculosis

## Abstract

Tuberculosis (TB) is a preventable and usually curable disease but remains a major health problem worldwide, particularly in developing countries. TB of the lumbosacral junction is rare and occurs in only 1–2% of all cases of spinal TB. Moreover, isolated sacrococcygeal TB is extremely rare. We present a case of a 64-year-old patient with a history of diabetes who presented with chronic back pain and cough. Physical examinations revealed a perianal fistula and left elbow joint arthritis. The patient is diagnosed with disseminated TB with coccygeal involvement. Diagnosis was achieved non-invasively using Xpert MTB/RIF, confirming *Mycobacterium tuberculosis* infection. The patient experienced complete resolution of symptoms following the commencement of anti-TB therapy. We highlight the importance of this case due to the rare coccygeal localization of TB in an immunocompetent patient, diagnosed through non-invasive means.

## Data Summary

No data were generated during this research or are required for the work to be produced.

## Introduction

Each year over 10 million people still fall ill with tuberculosis (TB) [1]. TB is an infectious disease caused by the bacterium *Mycobacterium tuberculosis*. While it primarily affects the lungs, leading to pulmonary TB, the infection can disseminate beyond the lungs through haematogenous and lymphatic spread, resulting in extrapulmonary tuberculosis (EPTB). EPTB refers to TB that affects organs other than the lungs. Around 15–25% of TB cases affect extrapulmonary sites, leading to EPTB through the haematogenous and lymphatic spread of *M. tuberculosis*. The most common sites of EPTB include the pleura, lymphatic system and osteoarticular structures [2].

Clinically, EPTB often goes underrecognized, with diagnoses frequently delayed due to its paucibacillary nature and atypical presentations. Factors such as Human Immunodeficiency Virus (HIV) infection and female gender have been identified as significant risk factors for the spread of EPTB. Diabetes is another critical factor, as multiple studies have shown it to be associated with a higher risk of developing active TB. Moreover, patients with diabetes have a greater risk of developing EPTB compared to pulmonary TB [3].

Disseminated TB is defined as a tuberculous infection involving the bloodstream, bone marrow, liver or two or more non-contiguous sites, or miliary TB. The symptoms are non-specific, and the duration of symptoms before diagnosis is variable.

Disseminated TB is a rare condition defined by the involvement of two extrapulmonary sites, with or without associated pulmonary involvement.

Osteoarticular TB represents between 1 and 3% of TB cases, with variable incidence in endemic and non-endemic areas of the world [4]. The lower thoracic and lumbar spine are the most common sites for spinal TB, whether it originates primarily or as a result of pulmonary TB. In contrast, infection of the lumbosacral junction is quite rare, occurring in only 1–2% of all spinal TB cases [5].

Here, we report a unique case of disseminated TB with rare sacrococcygeal involvement.

## Case report

A 64-year-old male patient presented with a 3-month history of persistent cough and severe back pain. The clinical history was notable for prolonged fever and significant weight loss. The patient had a history of type 2 diabetes; he did not have any past history of TB or any contact with another patient diagnosed with TB.

**Fig. 1.**
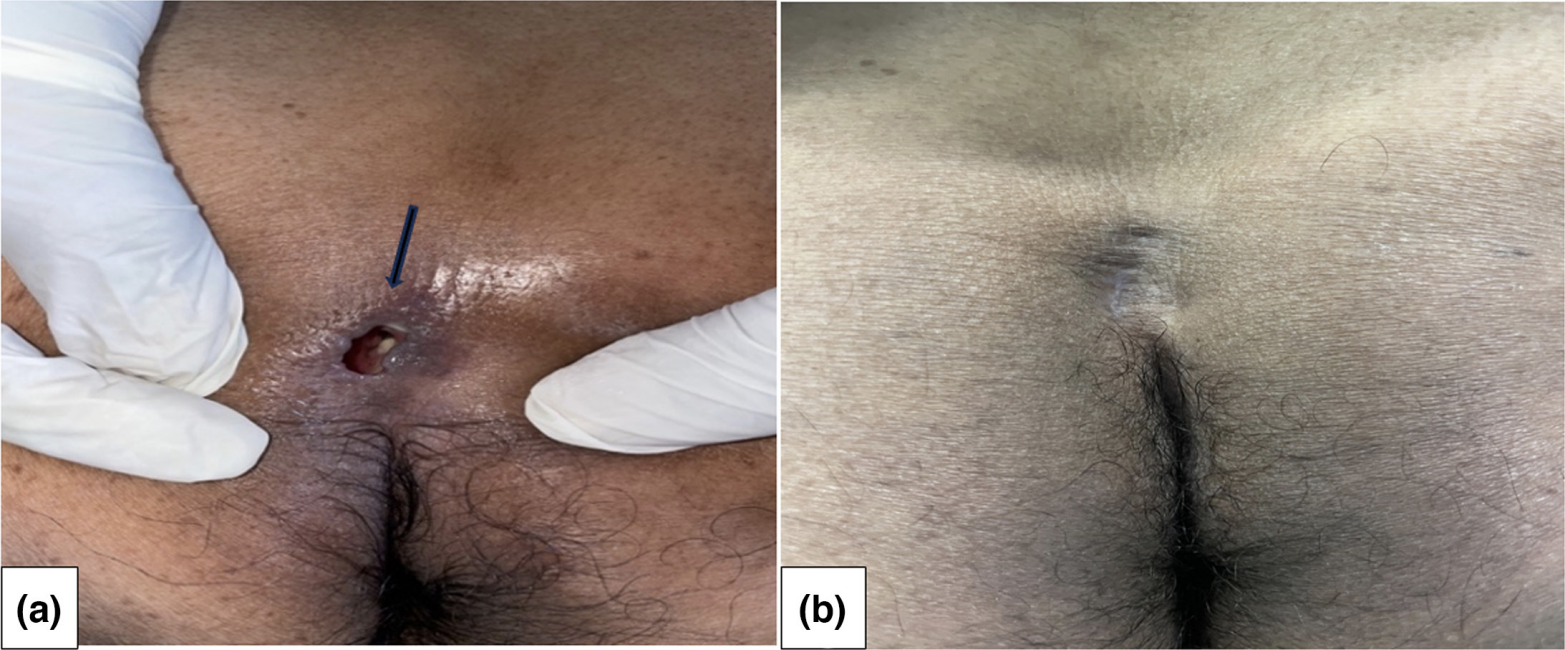
(a) Perianal fistula (see arrow) and (b) after 2 months of treatment.

The physical examination revealed a perianal fistula with purulent discharge (Fig. 1). The motor and sensory examination of the lower limbs was normal and there was no bowel or bladder incontinence. Osteoarticular examination found left elbow arthritis. The rest of the clinical examination was unremarkable.

**Fig. 2.**
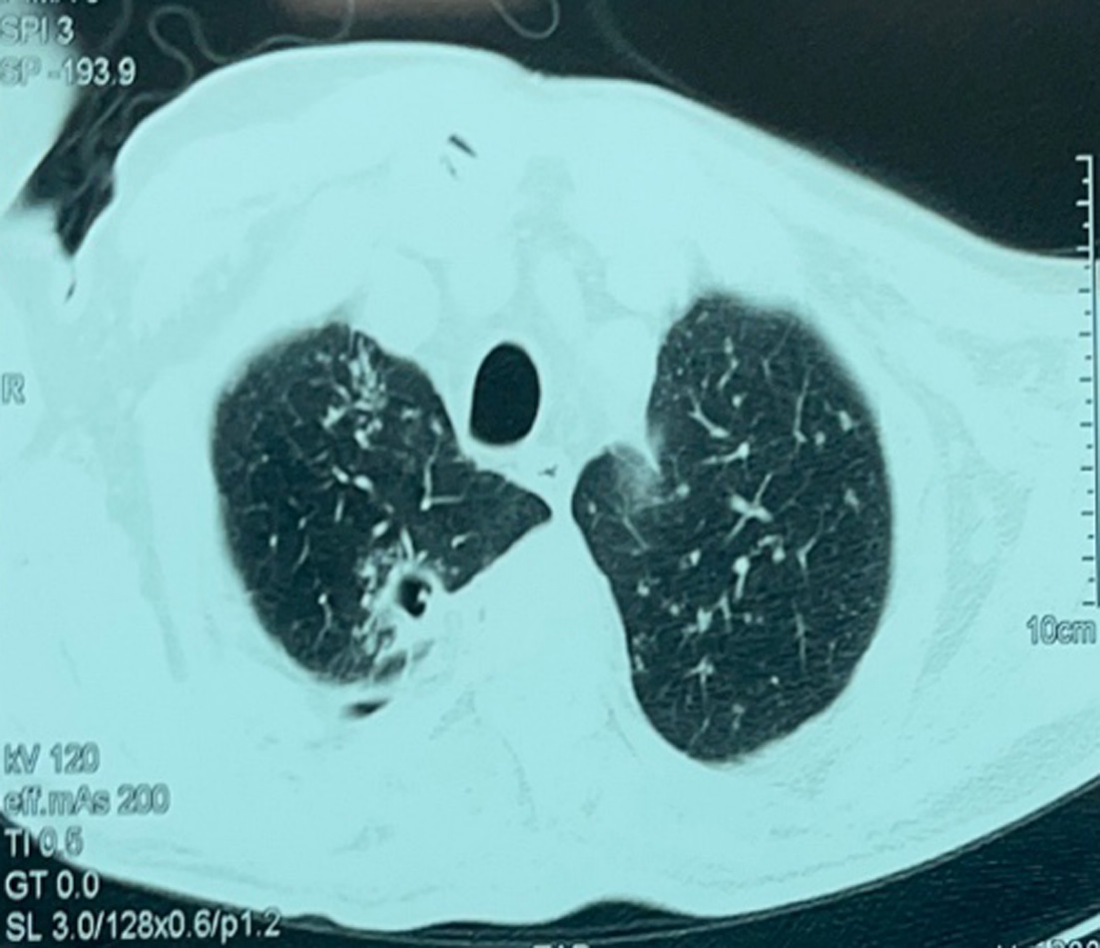
Chest CT scan shows consolidation with excavation (see arrow) in the right upper lobe.

**Fig. 3.**
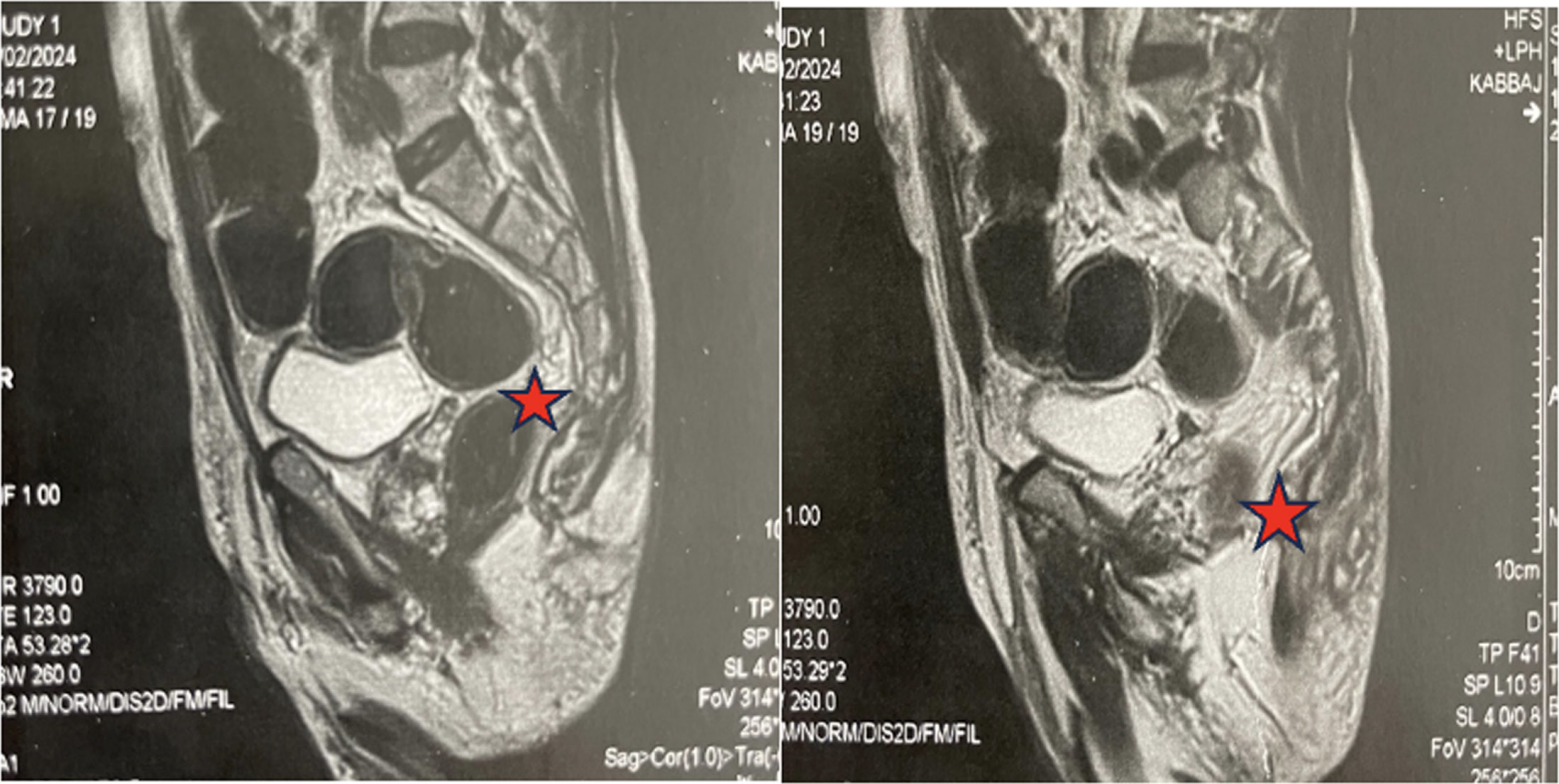
Pelvic MRI shows a pericoccygeal collection with soft tissue involvement and a fistulous tract.

**Fig. 4.**
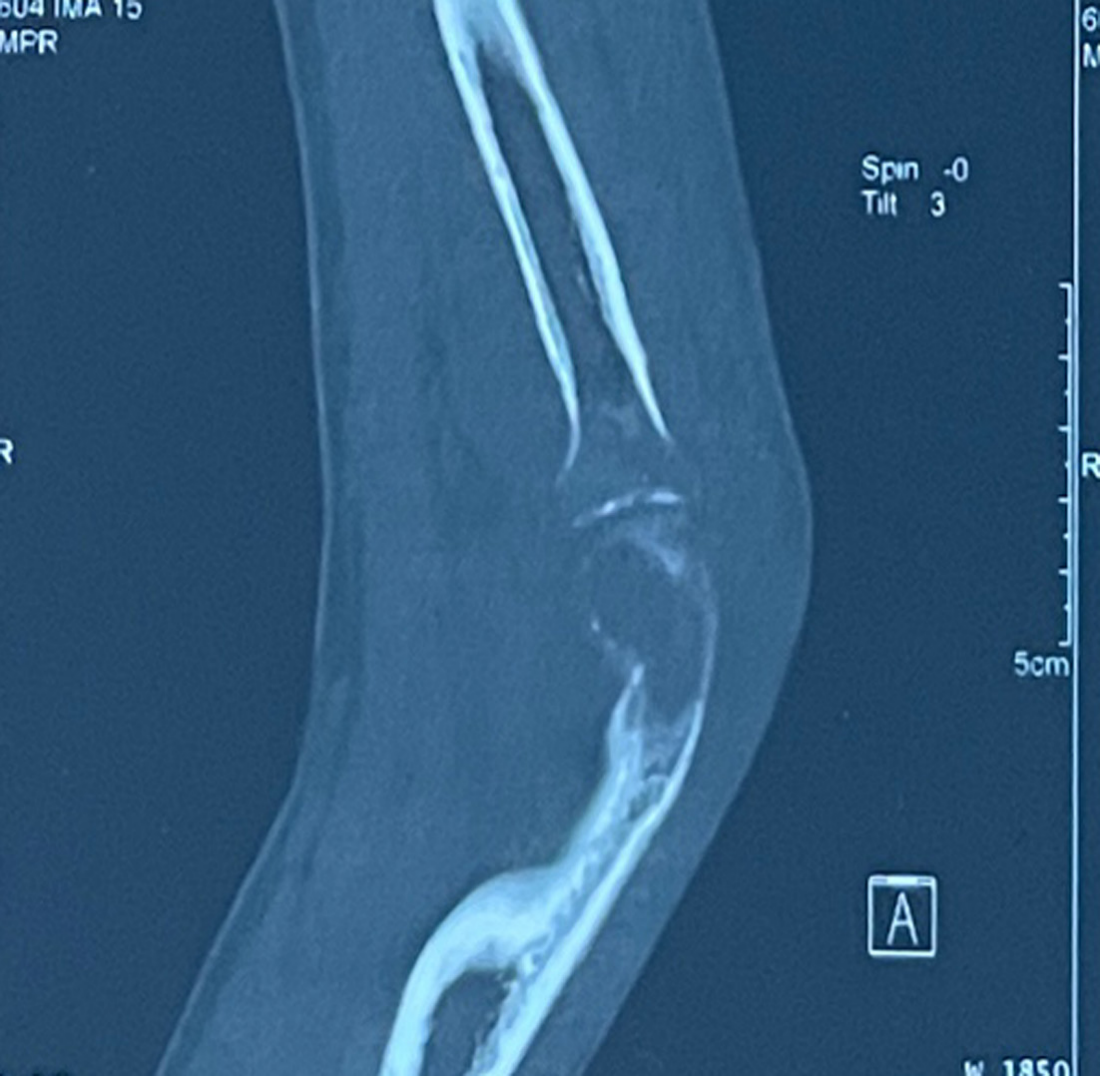
Left elbow CT scan shows joint effusion and severe osteopenia.

A thoracic, abdominal and pelvic computed tomography (CT) scan revealed a consolidation of the left upper right lobe with a cavity (Fig. 2), lysis of the D3-K3 costo-vertebral joint and the presence of coccygeal arthritis. Magnetic resonance imaging (MRI) of the lumbosacral spine demonstrates a complex lesion centred in the left median and paramedian retro-rectal space, extending into the pericoccygeal region. The lesion is enhanced in its periphery after gadolinium administration, and there is soft tissue involvement as well (Fig. 3). A CT scan of the elbow revealed a joint effusion with severe osteopenia (Fig. 4).

Blood tests showed elevation in C reactive protein level with normal blood cells. HBA1C=7%. He tested negative for HIV. The acid-fast bacilli (AFB) test performed on sputum and fistula discharge was positive. Additionally, the GeneXpert MTB/RIF assay conducted on sputum, fistula discharge and elbow joint fluid showed positive for *M. tuberculosis*.

We retained the diagnosis of multifocal TB with pulmonary and osteoarticular involvement of the left elbow and sacrococcygeal region, which is rare.

Antibacillary chemotherapy was started for 9 months. He was treated with rifampicin, isoniazid, ethambutol and pyrazinamide for 2 months, followed by rifampicin and isoniazid for 7 months, according to the recommendations of the national TB control programme in Morocco. There was no indication for surgical intervention. The patients' pain resolved and the purulent drainage stopped within the second month of treatment.

## Discussion

TB remains a global health concern posing significant challenges to both clinicians and radiologists due to its diverse and often non-specific clinical manifestations. Elbow TB is rare; its incidence varies from 2 to 5% of all skeletal locations [6].

In addition, TB infection of the lumbosacral junction is also quite rare, accounting for only 1–2% of all spinal TB cases [5]. Multifocal TB is defined by the occurrence of TB lesions in multiple locations across different adjacent or distant organs. Multifocal TB commonly occurs in immunocompromised patients, who differ from immunocompetent individuals in terms of clinical presentation, radiological features, laboratory findings and treatment outcomes, often leading to misdiagnosis [7].

We report a case of multifocal TB with atypical and rare localizations including the left elbow and sacrococcygeal joint. We were able to confirm our diagnosis at all sites using the GenXpert MTB/RIF test on the joint fluid, fistula discharge and sputum samples. Our patient is immunocompetent and has a history of well-controlled diabetes.

The diagnosis of EPTB can be challenging because of the diverse clinical presentations that mimic other medical conditions. Our patient reported initially elbow joint pain and back pain. Clinical manifestations of sacral TB depend primarily on patient age. Young individuals tend to present with discharging sinuses and abscesses, whereas backache is a dominant clinical feature in adults. Due to the protection provided by the sacral bone to the nerve roots, neurological symptoms in sacral TB are relatively uncommon [5]. Indeed, our patient does not present with any neurological symptoms, and the clinical examination shows no motor deficits or sensory dysfunction.

Only a few cases of coccygeal TB have been reported in the literature (see Table 1), and only three of them presented with sinus discharges. Concomitant active pulmonary TB is observed in around 50% of spine TB cases [8]. Our patient had active pulmonary TB as well with a positive AFB examination of the sputum.

**Table 1.** Sacrococcygeal TB documented in literature

Author	Country	Age	Gender	Presence of perianal discharge	Pulmonaryinvolvement	Diagnostic method
Thilakaranthe *et al.*, 2015	Sri Lanka	47	F	Yes	No	Biopsy under exploration of post-anal space
Kim *et al.*, 2012	Korea	35	M	No	No	CT guided biopsy
Singh *et al.*, 2011	India	20	F	No	No	Needle biopsy
Osman *et al.*, 2016	Tunisia	55	F	No	No	Surgery
Takakura *et al.*, 2018	Japan	93	M	Yes	No	Bacterial examination of fistula discharge
Kumar *et al*., 2006	India	42	F	Yes	No	Biopsy
Gadi *et al.*, 2019	India	23	F	No	No	CT guided biopsy

The usefulness of the GeneXpert MTB/RIF test is worth emphasizing due to its less invasive nature and high specificity for TB. The specificity of Xpert MTB/RIF for detecting TB is 99% [9].

Most cases of coccygeal TB have been diagnosed through invasive procedures such as biopsies or surgery, likely due to clinical presentations that often suggest alternative diagnoses, particularly malignancies. In our case, we confirmed the presence of * M. tuberculosis* using a rapid and non-invasive technique through PCR testing on various samples. The combination of immune, behavioural, anatomical and environmental factors likely contributed to the difference in presentation between our male patient and previously reported female cases. This unique presentation highlights the need for individualized consideration in the diagnosis and management of multifocal TB.

## Conclusion

Multifocal TB is an uncommon presentation of a disease that predominantly affects immunocompromised individuals. Our case, involving the rare combination of the elbow joint and sacrococcygeal region, highlights the diverse and atypical manifestations of this condition, emphasizing the importance of maintaining a high index of suspicion in unusual presentations. The use of molecular diagnostic tools such as GeneXpert played a pivotal role in confirming the diagnosis promptly, facilitating targeted treatment and improving the patient’s prognosis.

## Abbreviations

AFB: acid-fast bacilli
CT: computed tomography
EPTB: extrapulmonary tuberculosis
HIV: human immunodeficiency virus
MRI: magnetic resonance imaging
TB: tuberculosis
